# Transgenes in Mexican maize: molecular evidence and methodological considerations for GMO detection in landrace populations

**DOI:** 10.1111/j.1365-294X.2008.03993.x

**Published:** 2009-02

**Authors:** A PIÑEYRO-NELSON, J VAN HEERWAARDEN, H R PERALES, J A SERRATOS-HERNÁNDEZ, A RANGEL, M B HUFFORD, P GEPTS, A GARAY-ARROYO, R RIVERA-BUSTAMANTE, E R ÁLVAREZ-BUYLLA

**Affiliations:** * Laboratorio de Genética Molecular, Desarrollo y Evolución de Plantas, Dpto. de Ecología Funcional, Instituto de Ecología, UNAM, Tercer Circuito Exterior, Junto al Jardín BotánicoMéxico DF 04510, The Netherlands; † Biometric Applied Statistics, Wageningen UniversityPO Box 100, 6700 AC, Wageningen, The Netherlands; ‡ Departamento de Agroecología, El Colegio de la Frontera Sur, Carretera Panamericana y Periférico Sur s/n, San Cristóbal de las CasasChiapas, Mexico; § Universidad Autónoma de la Ciudad de México, Coordinación AcadémicaAvenida División del Norte 906, Narvarte Poniente 03020, México D.F., México; ¶ CINVESTAV Irapuato, Km. 9.6 Libramiento Norte, Carretera Irapuato-LeónA.P. 629, C.P. 36500 Irapuato, Guanajuato, México; ** Department of Plant Sciences/MS1, Section of Crop and Ecosystem Sciences, University of CaliforniaDavis, 1 Shields Avenue, Davis, CA 95616-8780, USA

**Keywords:** centers of origin, landraces, maize, Mexico, transgene flow

## Abstract

A possible consequence of planting genetically modified organisms (GMOs) in centres of crop origin is unintended gene flow into traditional landraces. In 2001, a study reported the presence of the transgenic 35S promoter in maize landraces sampled in 2000 from the Sierra Juarez of Oaxaca, Mexico. Analysis of a large sample taken from the same region in 2003 and 2004 could not confirm the existence of transgenes, thereby casting doubt on the earlier results. These two studies were based on different sampling and analytical procedures and are thus hard to compare. Here, we present new molecular data for this region that confirm the presence of transgenes in three of 23 localities sampled in 2001. Transgene sequences were not detected in samples taken in 2002 from nine localities, while directed samples taken in 2004 from two of the positive 2001 localities were again found to contain transgenic sequences. These findings suggest the persistence or re-introduction of transgenes up until 2004 in this area. We address variability in recombinant sequence detection by analyzing the consistency of current molecular assays. We also present theoretical results on the limitations of estimating the probability of transgene detection in samples taken from landraces. The inclusion of a limited number of female gametes and, more importantly, aggregated transgene distributions may significantly lower detection probabilities. Our analytical and sampling considerations help explain discrepancies among different detection efforts, including the one presented here, and provide considerations for the establishment of monitoring protocols to detect the presence of transgenes among structured populations of landraces.

## Introduction

Mexico is the centre of origin and diversification of maize (*Zea mays* L.) and is home to about 60 domesticated landraces (Sánchez *et al*. 2000) as well as several wild relatives with which domesticated maize can cross-pollinate (Doebley 2004). Maize is a staple food in Mexico, with a pivotal place in the country's past and present economic, cultural and agricultural spheres. In contrast to the United States and Europe, commercial seed sources account for only one-fourth of the maize seed planted in Mexico (Aquino *et al*. 2001). Furthermore, Mexican maize is mostly grown by smallholder farmers who obtain seed from their own harvest or from other farmers. This practice creates an open seed system, subject to evolutionary processes of drift, gene flow and selection, in which the fate of introduced transgenes is hard to predict (Bellon & Berthaud 2004). Therefore, a moratorium on field-testing and commercial planting of genetically engineered (GE) maize was established in 1998 in order to avoid unintended gene flow into local landraces and wild relatives.

In 2001, however, a study published in *Nature* reported the presence of the 35S Cauliflower Mosaic Virus (CaMV) promoter and nopaline synthase terminator (NOSt) recombinant sequences in four out of six ears sampled in 2000 from maize landraces in the Sierra Juárez region in the state of Oaxaca, Mexico (Quist & Chapela 2001). Although the paper was criticized due to its methodological shortcomings (Kaplinsky *et al*. 2002; Metz & Fütterer 2002), most critics did not argue with the paper's main conclusion, that transgenic elements could be present in their samples (Kaplinsky *et al*. 2002; Metz & Fütterer 2002). The concern about the introgression of transgenes into traditional landraces led to a number of biomonitoring efforts by Mexican governmental agencies, NGOs and research groups that differed both in methodology and results. These studies have been reviewed by Mercer & Wainwright (2008; see also Table S1, Supporting information). Peer-reviewed reports did not appear until 2005, when Ortíz-García and collaborators presented results from large samples of maize seeds collected in 2003 and 2004 from the Sierra Juárez region, that had been analyzed by two certified laboratories [Genescan, New Orleans, and Genetic ID (GID), Fairfield] detecting genetically modified organisms (GMOs). No evidence to support the presence of transgenes in the area was found, and Ortíz-García *et al*. (2005a) concluded that transgenes were either absent or extremely rare in the area. Although some published criticism of the sampling design and statistical analysis followed (Cleveland *et al*. 2005, see reply by Ortíz-García *et al*. 2005b), the issue of the presence and persistence of transgenes in Oaxaca has not been subsequently addressed with additional data and analyses.

The purpose of the present paper is twofold: (i) to contribute new data on the presence of transgenes in Mexican maize landraces; and (ii) to present an analysis of current molecular and sampling methods used in genetic monitoring that can help to explain contrasting detection results, while providing a clearer view of what the current limitations are for detecting transgenes in the field. Our molecular data corroborate the presence of transgenes in the Sierra Juárez region of Oaxaca in 2001 and their persistence (or re-introduction) up until 2004. Three out of 23 localities sampled by this study in 2001 were found to contain transgenes, while transgene sequences were not detected in another group of samples taken in 2002 from nine localities. However, directed sampling in 2004 of two of the localities that had yielded positive samples in 2001 again revealed the presence of transgenic sequences. We also provide data that suggest the sources of the false negatives, given the present-day molecular analytical methods used. Finally, we present theoretical results that show how sampling effects at the field level and, more importantly, clustered transgene distributions in structured maize populations, may significantly lower detection probabilities, thus helping explain current discrepancies among empirical studies.

## Materials and methods

### Collections

The 2001 maize collection reported here was conducted by CONABIO (National Biodiversity Council) and INE (National Ecology Institute) at 23 localities; two in Puebla, one in Southern Oaxaca and 18 from the Sierra Juárez of Oaxaca, as well as one seed market and a distribuidora conasupo, SA (DICONSA) store (in total 20 communities were sampled; see Table S2, Supporting information, for details). A single household was sampled in each locality. Samples consisted of one to five ears per household. All samples, except those from the DICONSA store, were confirmed to be landraces by owners and based on a visual inspection of ears or seeds by maize experts in our research group (H. Perales and J.A. Serratos-Hernández, personal communication). Samples from each location were blind-labelled, preserving location and maternal identity. An equal number of seeds sampled from each ear (from a total of 68, plus one bulked seed sample from DICONSA and one from a local market) were dispatched to the CINVESTAV (RRB) and IE UNAM (EAB) laboratories. Seed germination, DNA extraction and polymerase chain reaction (PCR) assays were conducted independently in each laboratory. A first set of independent PCR analyses yielded 10 localities having at least one seedling positive for the 35S promoter, as determined by both laboratories (locality codes: 2, 4, 5, 7, 10, 11, 14, 17, 21 and 23; please refer to Table S2 in the Supporting information for details). As seedling tissue and DNA were exhausted in this first round of analysis, a new set of seeds from the same ears were sown in each laboratory (EAB and RRB), and DNA was extracted anew from plantlets having five leaves or more in order to corroborate the existence of positive samples. Leaf material was stored for all plantlets and used in a second round of analyses. Samples were scored as positive for 35S transgene based on at least two independent DNA extractions and two positive PCR assays in each laboratory.

In 2002, maize seed samples were obtained from households as part of a socio-economic study of nine localities in the Sierra Juárez of Oaxaca and four of the municipalities previously sampled in this study: Ixtlán de Juárez, Santa Catarina Ixtepeji, Tlalixtac de Cabrera and Calpulalpan (in total 5 communities were sampled; see Table S3, Supporting information, for details). Furthermore, four out of the nine localities collected were the same as in 2001: 3, 10, 17 and 20, according to codes used for the 2001 collection (see Table S2 in the Supporting information for reference). Ears comprising all maize varieties cultivated within a household (hereafter called ‘seed lots’) were randomly sampled either from standing plants (9 ears/field) or from stored ears (6 ears/type). In all, 117 seed lots/fields were sampled, including a total of 682 ears.

In 2004, two localities from the Sierra Juárez region (locality 11 in Santiago Xiacui, and locality 7 in Santa María Jaltianguis; see Table S2, Supporting information) were sampled. In our analyses of 2001seedlings, these localities had consistently positive results for the 35S promoter sequence as determined by at least three independent PCR results per germinated plant, plus at least one positive Southern Blot (SB) hybridization. In each locality, 30 fields were sampled out of an estimated total of 50 fields per locality, and in each field, 300 leaves from randomly chosen plants throughout the field (95% probability of transgene detection at frequencies ≥ 1%) were collected. During leaf collection in each field, 4 mm leaf sections were bulked per field (totalling 300 sections per bulk). The remaining leaves were labelled and stored separately (a total of 9000 leaf samples per community were stored). The bulks comprising the 4 mm sections of 300 leaves each were subdivided into six bulks of 50 leaves each. DNA was extracted, and the presence of 35S and NOSt sequences was determined by PCR/assays. Data on maize seed history and management practices was collected in each household during the 2002 and 2004 collections.

### Molecular methods: sample preparation

For the 2001 collection, seeds were surface-sterilized (CAPTAN powder) and germinated under sterile dark conditions at 37 °C. Seedling coleoptiles and first true leaves were used for DNA extractions. For the 2004 collection, leaf sections of stored material were used. DNA was extracted from 6 cm^2^ leaf tissue by grinding the tissue in liquid N_2_in a mortar and pestle with 1000 µL of CTAB extraction buffer. For the 2002 samples, molecular analyses were performed directly on the collected maize seeds; a row of seeds along the length of every collected ear was sent for DNA extraction and PCR analyses at the GID and PG laboratories, respectively. For samples analyzed at GID, 20 seeds from each row of seeds from a maize ear collected at a particular locality were pooled together to form a sub-sample. The identity of each sub-sample was kept through individual packaging. Each sub-sample was sent to GID where it was ground separately. After grinding, an aliquot of flour was taken and bulked with flour from other sub-samples in order to form what was referred to as the main sample for DNA extraction and PCR analyses. The main sample was used to test for the amplification of the 35S and NOSt sequences. The maximum number of seeds represented in each main sample was 5100 (for details on number of seeds in each sub-sample, see Table S3, Supporting information). Samples analyzed by PG and collaborators were representative of the seed lot of a particular farmer; in this case, seeds from a seed lot were bulked and 25 seeds were randomly separated from this bulk. DNA was extracted and analyzed by PCR for presence of the 35S and NOSt sequences (see Table S3, Supporting information).

### DNA extraction and PCR amplifications

DNA extraction was adapted from CIMMYT Protocolos de laboratorio (CIMMYT, 2006). PCR products were run in 2% agarose gels stained with ethidium bromide. For PCR analyses conducted by PG, internal standards were prepared for the 2002 bulked seed samples using transgenic Syngenta GSS 0966 F1 maize (event BT11) and non-transgenic DeKalb DKC 62–15 maize. Standards containing 0, 1%, 5%, 10% and 100% transgenic seeds, which tested positive for both the 35S promoter and the NOSt down to the 1% level, were prepared. External standards were obtained from Fluka (now Sigma-Aldrich) and contained transgenic line DK 513 (event MON810) and non-transgenic DK 512 in ground flour at the level of 0, 0.1%, 0.5%, 1%, 2% and 5% flour of transgenic origin. These external standards only tested positive for the 35S promoter to the level of 0.1%; they were not tested for the NOSt because this sequence was lost during integration (review http://www.agbios.com for information about this event). The maize invertase gene (PG), and the maize HSP101 and 16S ribosomal units (EAB and RRB, respectively) were used as positive controls of the quality of DNA used for PCR amplification. In IE-UNAM and CINVESTAV-I, MON810, NK603 and the plasmid pMON18770 (provided by Monsanto® to RRB) were used as positive controls. We also conducted tests on a non-commercial CIMMYT maize transformation event expressing Cry1Ab, with an antibiotic resistance gene driven by the CaMV 35S promoter (Bohorova *et al*. 1999). All of the positive controls tested positive in all assays for the 35S promoter while the NOSt was present only in NK603. The NOSt was assayed in the 2002 and 2004 maize samples but not in the 2001 sample. A Peruvian landrace (sample 11 in Table 1) and a cross of two improved conventionally inbred maize lines from CIMMYT (CML-244 × CML-239; labelled as sample 12 in Table 1 and provided by JASH) were used as negative controls; these were negative in all assays. For the 2004 sample, we confirmed that at least one positive leaf section out of 50 could be detected by PCR.

**Table 1:** Molecular assays used to detect the presence of the CaMV 35S promoter and NOSt in a subset of leaf maize samples collected in 2001 from the Sierra Juárez, Oaxaca, Mexico. For the criteria to score presence (1) or absence (0) of the expected PCR band in the gels see text

		IE-UNAM		Genetic ID		
		PCR	SB	PCR		Qn-PCR
			
Locality	sample code	35S (N)	35S	35S	NOSt	35S
*Positive samples*						
*7*	1	1	1	1	1	0.60%
7	2	1	1	1	1	N.A.
7	3	1	0	1	1	N.A.
7	4	1	1	1	1	N.A.
7	5	1	—	1	1	100%
11	6	1	1	1	1	N.A.
23	7	1	1	1	1	N.A.
23	8	1	1	1	1	N.A.
23	9	1	1	1	1	N.A.
23	10	1	1	1	1	N.A.
*Negative controls*						
N.A.	11	0	0	0	0	N.A.
N.A.	12	0	0	0	0	N.A.
N.A.	13	0	0	0	0	N.A.
*Positive Controls*						
N.A.	MON810	1	1	1	N.A.	120%
N.A.	NK603	1	1	0	1	N.A.

PCR, Qualitative PCR; SB, Southern Blot; Qn-PCR, Quantitative PCR; N, Native Taq Polymerase; N.A., Not Applicable. We present only three of the 10 negative samples assayed.

PCR primers were: 35*S/*195/f *(*gene/size of PCR product/orientation): -GTCCCTACAAATGCCATCA; 35*S/*195/r-GATAGTGGGATTGTGCGTCA (Lipp *et al*. 1999); *NOSt/*118/f-GCATGACGTTATTTATGAGATGGG, *NOSt/*118/r-GACACCGCGCGCGATAATTTATCC (Lipp *et al*. 1999). All reported positive PCR reactions were based on at least three independent DNA extractions and independent PCR reactions with clear bands present in the second round of molecular analyses performed on plantlets from the 2001 collection and two independent PCR reactions for the 2004 collection (conducted at EAB laboratory).

### PCR analyses conducted by GID

**Fig. 1:**
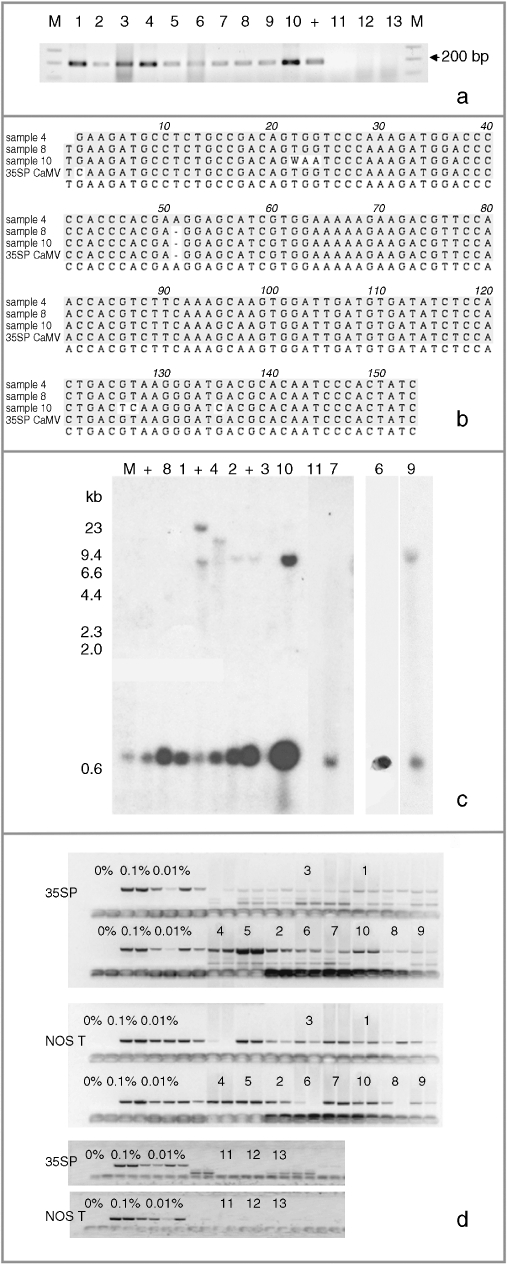
Molecular assays of individual leaf samples from 2001 landrace collections in Oaxaca. (a) PCR amplification of a 195-bp fragment of the CaMV 35S promoter done at EAB laboratory. (b) Sequence alignment of the three types of 195 bp PCR products for the CaMV 35S promoter found among the ten assayed individuals. (c) Southern blots (SB) for the same ten individuals as in (a), except sample 5. For all the samples shown in this SB, we have at least one other independent extraction and hybridization confirming either a positive (samples 1, 2, 4, 6, 7, 8, 9, 10 and 11) or negative (sample 3) result. Digested DNA was hybridized against a 639-bp probe containing a double 35S sequence. (d) GID PCR gels with duplicate PCR reactions for each assayed sample. To the left are positive controls: 0.1%; in duplicates and 0.01%; in quadruplicates. Only sample 5 was considered 100% positive by GID, but clear bands are present in all other positive samples. Sample labels correspond to the ones presented in Table 1. The positive DNA control in panel (a) is from corn NK603; positive controls in panel (c) are, from left to right: DNA from line MON810, and DNA from two separate seedlings of line NK603.

Leaf samples from individual plants and seed bulks sent for PCR reactions at GID were DNA extracted by this company in duplicate independent extractions. Afterwards, DNA from each extraction was subjected to independent PCR reactions for the internal DNA control gene and the 35S and NOSt sequences. PCR and primer details are not provided by GID for publication, but they use qualitative PCR reactions and primers specific for the 35S and NOSt sequences found in commercial maize lines. The relative percentage of transgenic sequences in a sample is estimated by visually comparing PCR electrophoresis gels with consecutive dilutions of positive controls (0.1% in duplicates and 0.01% in quadruplicates: see Fig. 1d) of the 35S and NOSt sequences. The negative control (marked as 0%) is the absence of DNA. As this company is certified to detect commercial transgenic sequences in bulked seed samples, the scoring criteria used in semi-quantitative PCR analyses consists of establishing the presence/absence of transgenic sequences according to the PCR band intensity, as well as an inspection of amplification consistency among duplicate samples. Afterwards, the relative percent of the marker under analysis is estimated in relation to the dilution series of the positive controls.

### Southern blot hybridization analyses

20 µg of genomic DNA was used per sample (except for sample 5 which did not hybridize in a single SB with only 10 µg of blotted DNA due to insufficient tissue). In order to release and identify by size, an insertion of a double CaMV 35S promoter present in 84% of the transgenic commercially available maize lines (according to http://www.agbios.com, verified 28 May 2008), DNA was digested with *Hin*dIII-*Bgl*II for 6 h at 37 °C. Thus, different intensities of hybridized bands among samples suggest different number of inserts per assayed genome. Blotting was performed according to Sambrook *et al.* (1989). The probe was obtained from pMON18770 using *Bgl*II and *Hin*dIII, labeled with ^32^P dCTPs with Amersham Bioscience Ready-To-Go DNA labelling beads and purified with Amersham Bioscience G-50 Sephadex columns according to the manufacturer's instructions. Positive and negative samples were included in each SB experiment and clear or no bands were apparent, respectively, in all tests.

### Simulating sampling efficiency in structured maize populations

The process of transgene introduction was modelled by simulating the population genetic dynamics of a neutral bi-allelic locus in a set of 1000 independent villages over time. Introductions of both heterozygous and homozygous individuals for the transgene were modelled. The most likely scenario is that heterozygous or hemizygous individuals are introduced, because this is the case in most commercial lines. The qualitative results are the same for both cases, but expected frequencies are somewhat lower in the former case. We present results for this more conservative scenario.

Each village was modelled as a square grid of 81 fields. Pollen flow was assumed to occur only between neighbouring fields and was set at 1.5% per synchronously flowering neighbour field (e.g. Messeguer *et al*. 2006). Seed migration was simulated as a complete or partial seed replacement with individual, randomly selected farmers as a source. Farmer interview data from two of the sampled localities was used to estimate the following model parameters: average population size (40 selected ears, 300 seeds per ear), one average neighbour with synchronized flowering and a replacement frequency of 0.07. Partial replacement (seed mixing) was assumed to occur with a frequency of 0.01 and involving 20 migrant ears. We modelled seven years of random introductions. The probability of planting a heterozygous transgenic seed lot in a single year was set at 0.01 per farmer. Farmers planting transgenic maize were excluded as a source for migration via seed and were set to abandon the seed in the next season. Detection probabilities for samples taken from the simulated frequency distribution were based on 10 000 random samples from 1000 simulated villages with *n*_*f*(*i*)_ fields per village, *n*_*c*(*i*,*j*)_ ears per field and *n_s_*_(*i,j*)_ seeds per field. The number of represented paternal alleles in a sample *n*_*u*(*i*,*j*)_ was set to *n*_*s*(*i*,*j*)_ in case of unrestricted mating. Restricted mating was imposed by setting *n*_*u*(*i*,*j*)_ to a reduced value that was generated by simulation of the pollination process using published data on flowering dynamics (Uribelarrea *et al*. 2002) and pollination patterns (Ma *et al*. 2004; see Appendix S1, Supporting information). The detection probability for each sampled field was thus given by: 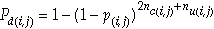 (see Appendix S1, Supporting information). Complete selfing was defined by a sample size of *n*_*c*(*i*,*j*)_ alleles. Sample sizes (i.e. number of villages, fields per villages, ears per field and seeds per ear) were set to reported values (Ortíz-García *et al*. 2005a; see also above).

## Results

Evidence for the presence of the 35S promoter was found by PCR and SB in localities 7, 11 and 23 from the 2001 collection. No transgenes were detected in 2002 localities. The latter did not include any of the localities with positive samples for the 35S promoter in 2001. For localities 7 and 11, a new directed survey and maize leaf collection was undertaken in 2004, and the presence of the 35S promoter was detected in 11 out of 60 fields.

### Analyses performed on the INE-CONABIO 2001 collection

From a first set of independent PCR analyses conducted in the EAB and RRB laboratories, 10 localities with at least one positive seedling for the 35S promoter in both laboratories were found (localities 2, 4, 5, 7, 10, 11, 14, 17, 21 and 23). In a second round of analyses, a total of 21 plantlets (out of 1867 analyzed plantlets) from three out of 23 localities (7, 11 and 23) were positive for 35S in two independent DNA extractions and PCR assays at the EAB and RRB laboratories. Thus, in this study, we considered these individuals and localities as confirmed positives, yielding an overall 0.011 (21/1867) frequency of transgenes in the total number of plantlets analyzed.

In order to further confirm the presence of the 35S promoter in the positive PCR plantlets, we selected 10 positive individuals at random from localities 7, 11 and 23 and performed additional independent DNA extractions and PCR assays at EAB, followed by SB hybridization assays for 35S (see Table 1 and Fig. 1). For locality 11, we had a very low germination rate and high mortality, and thus we could only attain enough tissue for one individual to be corroborated by SB (sample 6 in Table 1). An additional PCR assay yielded positive results for all of them (Fig. 1a), while positive SB results were obtained for only eight of the 10 samples (1, 2, 4, 6, 7, 8, 9 and 10; Fig. 1c).

We performed PCR and SB for a second group of plantlets that were considered to be negative controls. These plantlets had yielded at least three negative PCR results for 35S in the first round of analyses performed by EAB and RRB with the 2001 samples, or were controlled landrace samples known to be non-transgenic (provided by JASH laboratory). All of these samples yielded negative results in both types of molecular tests (see examples in Table 1 and Fig. 1).

We cloned and sequenced the PCR bands from the eight positive SB individuals, plus sample 5. We obtained three types of 35S CaMV promoter-like sequences (Fig. 1b). We present an alignment of three samples containing the three different sequences retrieved using the 35S promoter from the CaMV genome as a reference (GenBank Accession no: V00140). Sample 4 is identical to the reference sequence; sample 8 shows a 1-bp insertion while sample 10 differs by five nucleotide substitutions.

### Analyses performed in a 2002 collection

We sent an average of 20 seeds per ear, 14 343 seeds in total, to GID, and bulks of 25 seeds per seed lot were analyzed for 35S and NOSt by PCR at PG laboratory, totalling 2975 seeds. All samples were reported as negative and none of the gels for these samples showed visible PCR bands in either laboratory (Table S3 and Fig. S1, Supporting information).

### Confirming localized transgene presence in 2004

We conducted a directed sampling effort at two of the three localities that had yielded confirmed positives for the 35S promoter in 2001 (localities 11 and 7; see Table 2). We found three and eight positive fields out of 30 analyzed in localities 11 and 7, respectively. Therefore, 11 out of 60 fields collected in 2004 were positive for the 35S promoter sequence. This result was distributed among 14 positive 50-leaf bulks out of 360 bulks analyzed (see *Materials and methods*and Table 2).

**Table 2:** Number of bulks comprising leaf samples from localities 7 and ll in Oaxaca (2004) with a positive 35S PCR band. Thirty fields were collected per locality (labelled 1 to 30 for each one); 300 maize leaves were collected per field for molecular assays. Each 300-leaf bulks were disaggregated to 50-leaf bulks (6 per field) for DNA extraction and PCR amplification of the HSP101, 35S and NOSt sequences. Data on NOSt amplification not shown. Results presented here correspond to bulks that were confirmed as positive in at least two independent PCR reactions

Locality	Field	Number of 50 leaf bulks (total = 6 per field) positive for the 35S sequence
11	7	1
	17	1
	19	1
Positive fields in locality 11 = 3		
7	6	1
	8	1
	11	1
	15	1
	17	2
	25	1
	27	1
	30	3
Positive fields in locality 7 = 8		

### PCR results from a certified laboratory (GID)

We sent the 10 positive (the eight samples with positive SB, and samples 3 and 5 for which we had 35S sequences) and 10 of the negative samples (individually lyophilized leaf tissue) for PCR analysis at GID. While the positive samples yielded reactions with clear bands in gels and were thus scored as positives by us (see Table 1 and Fig. 1, panel d), none of the negative samples showed any bands. These negative controls were scored as negative by GID (see Fig. 1d for examples of negative samples). All of the positive samples were scored as above the level of detection, except sample 5, which yielded the strongest duplicate bands and was scored as 100% positive (see Table 1), confirming that this sample was indeed positive as had been indicated by our PCR assays and sequence data. For two samples from the 2001 collection (samples 1 and 5 in Table 1) and one positive control (MON810), real-time PCR was conducted in order to estimate the precise percentage of transgenic marker (35S) present. Sample 1 was scored as 0.60% positive, while sample 5 scored as 100% positive (reports available upon request). Finally, leaf tissue from MON810 transgenic maize line was scored as 100% positive and the event was identified correctly.

We tested for the occurrence of false negatives in GID by submitting several blind positive samples (Table 3). The company unequivocally detected 1/50, 1/100 and 1/1000 bulked seed samples that contained one transgenic seed (from the commercial MON810 event) in a pool of non-transgenic seeds. Also, GID was able to detect the presence of MON810 in a lyophilized leaf sample. However, GID reported that a lyophilized leaf sample from event NK603 was negative for 35S. When GID performed quantitative PCR analyses for the latter sample, it reported it as ‘above detection level’ but it could not be assigned as an unequivocal positive for 35S, as was the case for our positive landrace leaf samples (except sample 5). GID also reported that a leaf sample from the homozygous CIMMYT Cry1Ab transgenic line was negative; this is a line that had been confirmed to harbour a 35S promoter by the EAB laboratory (Bohorova *et al*. 1999). These results show that when sending leaf samples, false negatives do occur in the assays performed by GID (see Table 3; reports from GID that substantiate this data are available upon request).

**Table 3:** Molecular assays performed on blind positive samples sent to GID and used as positive controls for the presence of the CaMV 35S promoter

			IE-UNAM		*GID*	
			PCR	SB	PCR	Event detected
				
Event	Tissue	Bulk	35S	35S	35S	35S
MON810	Leaf	no	1	1	1	yes
NK603	Leaf	no	1	1	0	no
CIMMYT Cry1Ab	Leaf	no	1	1	0	N.A.
MON810	seed	1 in 50		N.A.	1	N.A.
MON810	seed	1 in 100	—	N.A.	1	N.A.
MON810	seed	1 in 1000	—	N.A.	1	N.A.

PCR Qualitative PCR; SB, Southern Blot; Qn-PCR, Quantitative PCR; N.A., Not Applicable. Scoring according to Table 1. The bulked seed samples reported in this table were not tested for in our laboratories, but plantlets from the same lot of MON810 seeds used in assays reported in this table tested positive in PCR reactions for the 35S sequence in both EAB and RRB laboratories, and also tested positive for Southern blot hybridization (in EAB laboratory). Lyophilized leaf tissue from lines MON810 and NK603, were used as positive controls in this study, and also listed in Table 1.

### Detection probabilities in landrace samples

Our experimental results suggest that the variability in the outcomes from the molecular assays may be a source of disagreement among detection efforts. However, as long as transgenes are rare in landrace populations, contrasting findings of presence/absence are more likely to be due to sampling effects. We discuss two aspects that affect transgene detectability that have thus far not been sufficiently considered. Our expectation of agreement between different samples depends on the detection probability, which is normally calculated as


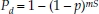
(eqn 1)

with *S* = *n* individuals or *S* = 2*n* alleles sampled per field, *m* sampled seed lots or fields, assuming that positive individuals are distributed with a uniform frequency *p* among sampled populations (Lockwood *et al*. 2007). A first limitation to the use of the above formula for field samples arises from the unequal paternal and maternal contribution to the sampled seeds. When sampling from a limited number of ears, it should be considered that half of the alleles from each ear derive from the same maternal plant (Cleveland *et al*. 2005). The number of seeds sampled per field, *S*, is hence an inappropriate measure of sample size. This may be dealt with by defining *p* as the *allelic* frequency and calculating sample size as the total number of independent alleles contained in each field sample. Assuming random pollination, this yields approximately *S* = 2*n_c_* + *n* (see Appendix S1, Supporting information), where *n_c_* is the number of ears sampled per field and *n* is the total number of seeds. This result means that for *n_c_* << *n*, *S* is close to *n* alleles, thus reducing the actual sample size by 50% compared to the 2*n* alleles assumed in eqn 1.

**Fig. 2:**
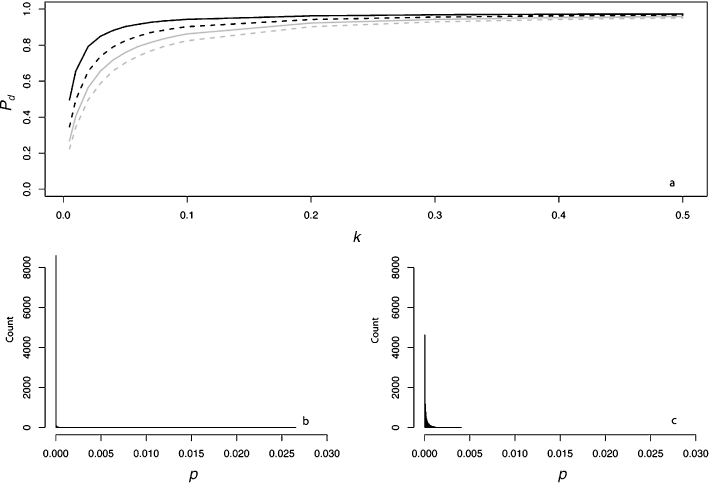
Effect of aggregated transgene distribution on the expected probability of detection (a). *P_d_* is shown over a range of values of the shape parameter of the gamma distribution (*k* = 0.005 : 0.5) at four different values of *m* (48, 24,16,12). Mean allele frequency *p*, and total sample size were set at 0.0002 and 24 000, respectively. Lower panels show histograms of 10 000 random values of *p* at *k* = 0.03 (b), and *k* = 0.35 (c).

Secondly, the assumption of uniform frequencies is likely to be violated, because transgenes are presumably introduced locally by individual farmers. The effect of transgene frequency differences on *P_d_* is not, however, obvious. Local aggregations of transgenes lead to higher detection probabilities in those sites, offsetting the decreased probability in fields where transgenes are rare. We may approximate the overall detection probability in a sample taken from *m* fields with frequencies *p_i_* by: *P_d_* ≈ 1 − ((1 − *p*)^*S*^Ψ)^*m*^ with 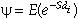 and *d_i_* = *p_i_* − *p* (Appendix S1, Supporting information). Numerical analyses reveal that Ψ is close to unity for high levels of aggregation and *P_d_* is correctly estimated by eqn 1. However, for extremely skewed frequency distributions and high values of *S*, Ψ increases and detection probabilities decrease considerably (Fig. 2). In these cases, individual fields can be said to be over-sampled.

We may estimate the expected level of transgene aggregation by simulating the process of unintentional introduction over time, using data on pollen and seed flow (see *Materials and methods* and Appendix S1, Supporting information). Indeed, the simulated frequency distribution was highly skewed (Appendix S1, Supporting information, and Fig. 2), with most fields having frequencies close to zero and few having frequencies of over two percent. Evidently, pollen and seed flow did not homogenize transgene frequencies among populations. A comparison of detection probabilities for samples taken from this distribution can be seen in Table 4. Results are presented for our 2001 and 2002 collections as well as for data from Ortíz-García *et al*. (2005a) regarding their 2003 and 2004 samples. Values are shown for unrestricted pollination, restricted pollination and complete selfing. Detection probabilities under the assumption of uniform frequency across fields are provided for comparison. Except in the case of complete selfing, aggregated transgene frequencies clearly reduced the detection probability in the different studies.

**Table 4:** Detection probabilities (*P_d_*) of transgenes in three independent samples from a simulated transgene distribution based on sample sizes used in two studies conducted in the Sierra Juárez, Oaxaca (México). See text for details

		*P_d_* uniform frequency			*P_d_* simulated aggregation		
Study	Mean allelic freq	Unrestricted pollination	Restricted pollination	Complete selfing	Unrestricted pollination	Restricted pollination	Complete selfing
2001	0.0002	0.33	0.28	0.01	0.22	0.21	0.01
2002	0.0002	0.99	0.97	0.13	0.87	0.85	0.12
2003*	0.0002	1.00	0.99	0.03	0.82	0.65	0.03
2004*	0.0002	1.00	1.00	0.14	0.94	0.92	0.14

* sample from Ortíz-García *et al.* 2005a.

## Discussion

### Confirmation of the presence of the 35S promoter in a 2001 sample and consistency of different molecular assays

We have provided new, unequivocal evidence that transgenes were present in Oaxaca in 2001. Our data suggest that transgenes were present in our sample at a frequency of 0.011 based on PCR, and at a frequency of 0.0089 based on SB hybridization. We do not claim that the sample frequency is a reliable estimate of actual transgene frequencies in the field. Given the expected aggregated frequency distributions, it would be misleading to take sample frequencies based on a small number of seed lots per locality as a population estimate.

The consistency of the results among PCR replicates and Southern analysis suggests that PCR assays performed on individual leaves are a reliable method for transgene detection, provided that adequate positive and negative controls are used. The possibility of confirming PCR results by using multiple tests on independent DNA extractions from leaf tissue from the same individual renders the use of this type of material preferable over the use of seeds that are used up in a single assay. For this reason, it is important to conduct additional molecular analyses that will help establish the consistency of the results obtained for the same individual, by using different molecular assays or other laboratories.

The facts that all positive samples for both PCR and SB yielded clear positive bands in GID gels, while the negative samples in our laboratory did not show any bands in this commercial laboratory, confirm that consistent positive PCR results are accurate and that SB may yield false negatives, especially when a small amount of DNA is used. For example, the facts that sample 5 was consistently positive by PCR, and that its product was sequenced and confirmed to be 35S, suggest that the negative SB result when using only 10 µg of DNA is a false negative. Indeed, other samples that were confirmed to be positive with SB when using 20 µg of DNA were negative when less DNA was blotted (data not shown). On the other hand, we did obtain false positives in isolated PCR assays. Therefore, we considered as positive only those plantlets with at least three positive assays from independent DNA extractions and PCR reactions. In contrast, SB assays, while prone to false negatives, never yielded false positives. Hence, when using the optimum amount of DNA, SB is a powerful confirmatory tool.

### Analysis by GID

GID confirmed (either as 100% positive or with a clear detectable band) the presence of 35S in all the samples sent that had tested positive by PCR in our laboratory, suggesting that this company can detect transgenes in leaf tissue from landraces. However, we have shown that their conservative criteria for officially declaring a sample as positive can lead to reporting false negatives. In contrast to the criteria used in research-oriented laboratories, GID has set their criteria according to assays based on bulked seed samples, where it claims it is able to detect at least one in up to 10 000 seed bulks. Therefore, GID's scoring criterion was difficult to reconcile with the results obtained for PCR assays based on the lyophilized leaf tissue of individual maize plants. All samples present in Fig. 1d, except for sample number 5, were originally scored by GID as negative (‘not detected’), despite clear amplification of the expected fragment. The amplification efficiency was thought to be too low for a 100% (homozygous) or 50% (heterozygous) transgenic plant, based on the laboratory's experience (although no PCR amplification of a 100% positive control is ordinarily run by GID in its PCR tests). Once we acknowledged that the sent samples were from individual plants, posterior analyses of samples that presented this pattern of amplification were scored as detectable (‘detected’), but no estimation of the concentration of the transgenic marker analyzed was provided.

We scored all of the samples with clear amplifications (10) as positive, according to presence of expected bands in electrophoresis gels provided by GID (Table 1 and Fig. 1d). However, if we had maintained initial scoring used by GID, 90% of our positive samples would have been rendered as ‘above detection level’ due to the clear bands shown (Fig. 1d) but officially reported as negative due to PCR-band intensities below the minimum expected threshold.

While the change in criteria for reporting a sample as positive or negative in the case of lyophilized leaf tissue can aid in receipt of more biologically accurate reports, we did find evidence that GID assays seem to be prone to false negatives when using lyophilized leaf tissue. For instance, two of the three blind positives sent to GID were reported as negative (see Tables 1 and 3). While one of these blind positives consisted of a non-commercial transgenic event that had been confirmed to have a 35S promoter, GID also failed to correctly detect the transgenic markers and to establish the identity of the NK603 commercial event. Leaf tissue from the same positive control plants, MON810 and NK603 was used for the PCR and SB analyses performed at our laboratories and was revealed in several panels to be clear positives (Fig. 1). In conclusion, further standardization and a change in scoring criteria should be considered by certified GMO testing laboratories, such as GID, when performing PCR analyses on lyophilized leaf tissue and/or non-commercial events.

Standardization of PCR reactions on DNA from lyophilized leaf tissue may prove to be a complex task. In our laboratory, the intensity of the 35S PCR amplifications using standardized methods also varied among reactions even for confirmed positive samples with tested DNA quality (Fig. 1a). We currently have no explanation for the observed low amplification yields of the 35S promoter in some reactions and not in others. One possible cause includes inefficient primer binding due to event-specific modifications of 35S and NOSt priming sequences for which the current protocols are optimized (Anklam *et al*. 2002 and references therein). DNA quality and interference of plant metabolites could be other influencing factors.

### Transgenes absent in 2002 and persistent/reintroduced in a directed 2004 sample

In order to obtain evidence of transgene persistence, we re-sampled the same geographical region (Sierra Juárez) in 2002 and 2004. Our 2002 bulked seed samples showed no evidence for transgene presence in Oaxaca. In contrast, a directed sampling of a large number of fields (60) from two of the three localities that yielded positives in 2001 (localities 7 and 11 in Table S2, Supporting information) provided evidence for local transgene persistence or reintroduction up until 2004. Further analyses are needed to assess if introgression and/or repeated introduction have taken place, as well as to obtain accurate frequency estimates of transgenes in local maize races. Reintroductions are unlikely, however, given that none of the sampled seed lots were reported as having been derived from non-local seed. Therefore, our results suggest that once introduced, transgenes may persist at detectable frequencies within local maize populations, as was recently documented for wild *Brassica napa* and bentgrass (Reichmann *et al*. 2006; Warwick *et al*. 2008; Zapiola *et al*. 2008). This phenomenon is certainly possible given the documented and quantitatively modelled dynamics of maize seed exchange in Mexico (Dyer & Taylor 2008).

### Interpreting differences between detection efforts

Ortíz-García *et al*. (2005a) proposed two main explanations for their inability to confirm the presence of transgenes in the Sierra Juárez region in 2003–2004: either a possible reduction in transgene frequency from 2000 to 2003, or the absence of transgenes in 2000. Our study confirms that the 35S promoter was present in landraces in 2001 and that transgenes have probably persisted or may have been reintroduced to detectable levels in some of the localities, at least until the Autumn of 2004. Our 2002 results are in line with those presented by Ortíz-García *et al*. (2005a). We are thus faced with varying outcomes from different detection efforts in the same region.

Two factors may contribute to variation in the detection results. First, we need to consider the possibility of false negatives when transgenes are actually present in the sample. Ortíz-García *et al*. (2005a) mentioned the failure of PCR detection assays due to random errors in analytical procedure as a possible, although unlikely, source of false negatives. Our results show that false negatives do indeed occur when analyzing lyophilized leaf tissue. This finding is important because, unlike seed samples, leaves may be used for the subsequent confirmation of detection results by different laboratories and different assay techniques. Our limited results on seed-based detection by GID provide no basis on which to suspect that false negatives occurred in the analysis performed on seed bulks by both Ortíz-García *et al*. (2005a) and ourselves. However, in light of the rather unexpected variability in leaf-tissue results we do feel that a more thorough analysis of false negatives is called for. We recommend evaluating current quantitative PCR assays using different types of landrace seed to assess the possibility that chemical and/or genetic compositions that deviate from those that have been used to establish current detection limits may affect test outcomes.

Assuming that false negatives are rare, sampling effects remain as the most likely source of differing detection results. This aspect of sampling was considered by Ortíz-García *et al*. (2005b), and they corrected their estimates of reduced sample size within fields caused by the unequal maternal and paternal contributions to seed. Although their approach is conservative at the field level, it fails to account for the reduction in detection probability due to the expected skewed frequency distribution of transgenes in the study area. Our simulation results show that although unequal gametic contribution affects detection probabilities, the strongest reduction is due to the over-sampling of individual fields when frequencies differ strongly among them. Thus, it is important to stress that our simulations were to some extent conservative in that they assumed the immediate extinction of introduced transgenes. The persistence of primary introductions would yield an even greater heterogeneity of transgene frequencies and lower detection probabilities for a given mean frequency. Therefore, we propose that any confirmation of earlier findings should take place through extensive sampling of the same localities that previously yielded positive samples. This proposition is strengthened by our positive PCR results in 2004, which were obtained from larger samples within two of the communities in which transgenes had been detected in 2001.

The model parameters used here are based on survey data gathered for this study in a particular region of Mexico. However, these parameters are likely to vary across different maize agricultural systems. Future studies should therefore analyze the effect of contrasting production and seed management conditions on transgene frequency distributions and their detection probabilities.

This study has confirmed transgene presence in 2001 and 2004 in landraces from the Sierra Juárez region in Oaxaca, Mexico. Our results further suggest that transgenes are present at relatively low frequencies. Given the uncertainties revealed in the present work, however, more research is needed to allow for the reliable estimation of transgene frequencies in maize landraces. Future studies should provide precise estimates of local transgene frequencies from 2004 onwards. These studies are needed in order to monitor the persistence and frequency change of transgenes, both in Oaxaca and other parts of Mexico, where landrace maize samples with transgenic markers have been reported (Serratos *et al*. 2007), as well as in other parts of the country where no systematic studies have been undertaken. At present, we may only conclude that the failure to detect transgenes in individual studies should not be taken as evidence of their absence based on the sampling and analytical methods used up to now. Unintended transgene flow into Mexican maize landraces has been confirmed in this paper, and thus it is urgent to establish rigorous molecular and sampling criteria for biomonitoring at centres of crop origination and diversification.

## Notes

A. Piñeyro-Nelson is interested in molecular genetics and plant evolutionary development. J. van Heerwaarden is interested in the evolutionary genetics of domesticated plants. H. Perales studies the diversity and recent evolution of maize landraces in Mexico. J.A. Serratos-Hernández studies maize genetic resources conservation and biosecurity issues. A. Rangel has been involved in GMO detection services. M.B. Hufford is interested in evolution of invasive species and biodiversity. P. Gepts studies the evolutionary processes that have shaped evolution of crop plants under cultivation. A. Garay-Arroyo is interested in the genetic and molecular bases of plant development. R. Rivera-Bustamante is interested in plant virology and diagnostic procedures. E.R. Alvarez-Buylla is interested in genetics, evolutionary development and plant conservation as well as biomathematics.

## Supporting Information

**Appendix S1** Estimation of sample size and detection probability calculations

**Fig. S1** PCR analyses performed by GID on maize seed samples from the 2002 collection.

**Table S1** Comparison of experimental conditions and results of the various experiments discussed or reported in this study

**Table S2** Localities sampled in the 2001 Collection (INE-CONABIO)

**Table S3** 2002 collection results. Localities and seeds samples analyzed at GID and UCD are listed

## References

[b1] Anklam E, Madani F, Petra H (2002). Analytical methods for detection and determination of genetically modified organisms in agricultural crops and plant-derived food products. European Food Research and Technology.

[b2] Aquino P, Carrión F, Calvo R, Flores D, Pingali PL (2001). Selected Maize Statistics. CIMMYT 1999–2000 World Maize Facts and Trends, Meeting World Maize Needs: Technological Opportunities and Priorities for the Public Sector.

[b3] Bellon M, Berthaud J (2004). Transgenic maize and the evolution of landrace diversity in Mexico: The importance of farmers’ behavior. Plant Physiology.

[b4] Bohorova N, Zhang W, Julstrum P (1999). Production of transgenic tropical maize with cryIAb and cryIAcgenes via microprojectile bombardment of immature embryos. Theoretical and Applied Genetics.

[b5] CIMMYT (2006). Protocolos de laboratorio. http://www.cimmyt.org/spanish/docs/manual/protocols/Lab-geneticaMolecular.pdf.

[b6] Cleveland DA, Soleri D, Aragón-Cuevas F (2005). Detecting (trans) gene flow to landraces in centers of crop origin: lessons from the case of maize in Mexico. Environmental Biosafety Research.

[b7] Doebley J (2004). The Genetics of Maize Evolution. Annual Review of Genetics.

[b8] Dyer JA, Taylor JE (2008). A crop population perspective on maize seed systems in Mexico. Proceedings of the Nacional Academy of Sciences.

[b9] Kaplinsky N, Braun B, Lisch D (2002). Maize transgene results in Mexico are artefacts. Nature.

[b10] Lipp M, Brodmann P, Pietsch K (1999). IUPAC collaborative trial study of a method to detect genetically modified soy beans and maize in dried powder. The Journal of AOAC International.

[b11] Lockwood DR, Richards CM, Volk GM (2007). Probabilistic models for collecting genetic diversity: comparisons, caveats, and limitations. Crop Science.

[b12] Ma B, Subedi KD, Reid L-M (2004). Extent of cross-fertilization in maize by pollen from neighboring transgenic hybrids. Crop Science.

[b13] Mercer KL, Wainwright JD (2008). Gene flow from transgenic maize to landraces in Mexico: an analysis. Agriculture, Ecosystems and Environment.

[b14] Messeguer J, Peñas G, Ballester J (2006). Pollen-mediated gene flow in maize in real situations of coexistence. Plant Biotechnology Journal.

[b15] Metz M, Fütterer J (2002). Suspect evidence of transgenic contamination. Nature.

[b16] Ortíz-García S, Ezcurra E, Schoel B (2005a). Absence of detectable transgenes in local landraces of maize in Oaxaca, Mexico. Proceedings of the National Academy of Sciences.

[b17] Ortiz-García S, Ezcurra E, Schoel B (2005b). Reply to Cleveland *et al*.'s ‘Detecting (trans)gene flow to landraces in centers of crop origin: lessons from the case of maize in Mexico.’. Environmental Biosafety Research.

[b18] Quist D, Chapela I (2001). Transgenic DNA introgressed into traditional maize landraces in Oaxaca, Mexico. Nature.

[b19] Reichmann JR, Watrud SL, Henry Lee E (2006). Establishment of transgenic herbicide-resistant creeping bentgrass (Agrostis stolonifera L.) in nonagronomic habitats. Molecular Ecology.

[b20] Sambrook J, Fritsch EF, Maniatis T (1989). Molecular Cloning a Laboratory Manual.

[b21] Sánchez GJ, Goodman MM, Stuber CW (2000). Isozymatic and morphological diversity in the races of maize of Mexico. Economic Botany.

[b22] Serratos-Hernández JA, Gómez-Olivares JL, Salinas-Arreortua N (2007). Transgenic proteins in maize in the soil conservation area of the Federal District. Frontiers in Ecology and the Environment.

[b23] Uribelarrea M, Carcova J, Otegui ME, Westgate ME (2002). Crop Science.

[b24] Warwick SI, Lêgeré A, Simard MJ, James T (2008). Do escaped transgenes persist in nature? The case of an herbicide resistance transgene in a weedy *Brassica rapa* population. Molecular Ecology.

[b25] Zapiola ML, Campbell CK, Butler MD, Mallory-Smith CA (2008). Escape and establishment of transgenic glyphosate-resistant creeping bentgrass *Agrostis stolonifera* in Oregon, USA: a 4-year study. Journal of Applied Ecology.

