# Supplementary material for: Transgenes in Mexican maize: molecular evidence and methodological considerations for GMO detection in landrace populations
Source: Mol Ecol. 2009 Feb;18(4):750–61. doi: 10.1111/j.1365-294X.2008.03993.x (PMC3001031; doi:10.1111/j.1365-294X.2008.03993.x)
Supplement: Supplementary file 1 [file mec0018-0750-SD1.doc]

**Appendix S1: Estimation of Sample Size and Detection Probability Calculations**

**Sample size.** The detection probability, $P_{d}$, for samples of fixed size taken from different populations among which the transgene is distributed uniformly, is given by (Lockwood et al. 2007):

(1)

Where $m$ is the number of sampled seed lots or fields, $p$ the frequency of individuals containing the rare allele and $S$ is the sample size defined as the number of individuals collected per field.

It was recently argued that the number of individuals is not an appropriate measure of sample size. When seed is harvested from a limited number of ears, the unequal contribution of paternal and maternal parents has to be taken into consideration (Cleveland et al. 2005). The authors proposed replacing$S$ in equation (1) with the variance effective population size $N_{e}$. This measure is related to the variance of the allele frequency in the sample such that . It can be written as a function of the number of maternal and paternal parents as: (Vencovsky et al. 1999). Where $n_{c}$ is the number of maize ears and $n$ the total number of seeds sampled. When , $N_{e}$ reaches a maximum value at $4n_{c}$, so $S$ is at most four times the number of sampled ears.

We agree with the need to adjust sample size for unequal contribution of paternal and maternal alleles. However, we note here that effective population size is not informative of transgene detection probability. Variance in allele frequency between samples, and hence $N_{e}$, is dominated by $n_{c}$ due to the genetic correlation of seeds from the same ear. Samples containing ears derived from transgenic maternal plants will have very high transgene frequencies, thereby increasing frequency variance. As transgenes are expected to be rare however, most samples will contain no transgenic plants. Hence, the probability of finding a transgene is largely determined by the chance of occurrence in $n$ independent pollination events. presents this graphically by showing the distribution of the transgenic allele in 10,000 simulated ear samples from a single population with a transgene frequency of 1%. The sample frequency has a variance of 0.00026, which is as expected given the effective population size of 19.7 ($n_{c}=5$, $n=1500$). The distribution has a long right tail due to the sampling of positive ears. Three separate distributions can be observed.

These correspond to seed derived from negative homozygous maternal plants, positive heterozygotes, and positive homozygotes respectively. Negative homozygotes are the most frequent maternal plants, so transgene frequency in most samples falls within the narrow distribution on the left. Using the effective population size of *N_e_* = 19.7 = 39.4 alleles yields: . In contrast, all 10,000 samples contained at least one transgenic allele. This shows that $N_{e}$, although it accurately predicts the variance of allele frequencies in a sample, is not a proper measure of sample size when detection probability is the measure interest.


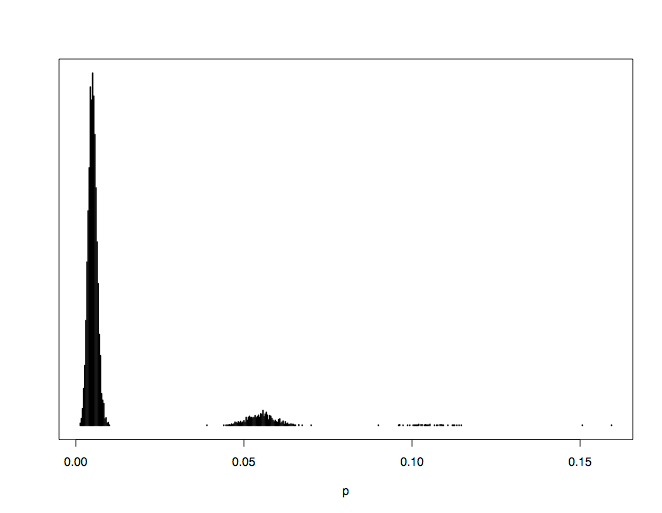


Figure 1. Histogram showing the distribution of transgene frequency in 10,000 simulated samples consisting of 5 ears and 1500 seeds. Mean frequency in the population 0.01. Variance corresponded to that expected from effective population size (0.00026, 2Ne= 39.4). Expected probability of non-detection using Ne was 0.67. No samples without transgenes were found.

For the purpose of transgene detection, sample size should be calculated as the number of independent maternal and paternal alleles represented in a sample. As was pointed out by Crossa (1989), determining the number of parents in a sample is a classical occupancy problem. In our case we define a single population of $N$ parental plants, containing $2N$ alleles. A sample of $n$ seeds taken from these plants will contain $S_{a}$ alleles that are represented at least once in the sample:

where $S_{m}$ is the number of paternal alleles and $S_{f}$ the number of maternal alleles.

We assume an infinite amount of pollen and random mating. The probability of including any paternal allele is:

The expectation of the number of alleles represented in the sample thus becomes (Crossa 1989):

When this equation approaches:

A sampled ear yields $n_{s}$ seeds such that $n=n_{s}n_{c}$. For maternal alleles we need to account for the fact that $n_{s}$ seed from the same ear will contain only one or two maternal alleles. The probability of having only one allele represented is given by:

The expectation for the number of alleles in a sample of $n_{c}$ ears thus becomes:

Under the assumptions mentioned above we may thus simplify $S_{a}$to:

(3)

Assuming , we may consider $S_{a}$ a sample with replacement from the total set of $2N$ alleles. Defining $p$ as the allele frequency of the transgene, we may substitute $S_{a}$ for $S$. $S_{a}$ may be adjusted for restricted pollination by replacing $n$ with the number of represented paternal alleles as estimated by the pollen simulation described below.

**Simulation of pollination process.** The distribution of paternity of seeds sampled from a single ear was simulated as a spatially explicit, competitive sampling process determined by flowering synchronicity between male and female inflorescences and distance between plants. A field of N = 60,000 plants was modeled assuming three plants per hill and 0.75 m between hills (Figure 2). Each plant was randomly assigned an anthesis and silking date based on actual field data (Van Heerwaarden, unpublished data). Data on day-to-day silk emergence and pollen production were derived from the study by Uribelarrea et al. (2002) and a total of 505 silks were assumed to emerge in discrete groups over 7 days. Silks emerging on a single day were assigned pollen parents by drawing with replacement from a probability vector representing the entire set of plants. Probability of paternity *p_i_* for each plant was defined as follows ,

where *G_i_* is the amount of pollen produced by plant *i* on that day and $d_{i}=e^{-0.4098x}$ representing the reduction of pollen concentration with distance *x* (Ma et al. 2004). The mean number of unique paternal alleles *n_u_* in a sample of *n_s_* seeds was determined by drawing samples of size *n_s_* from 100 simulated vectors of sires (Figure 3).


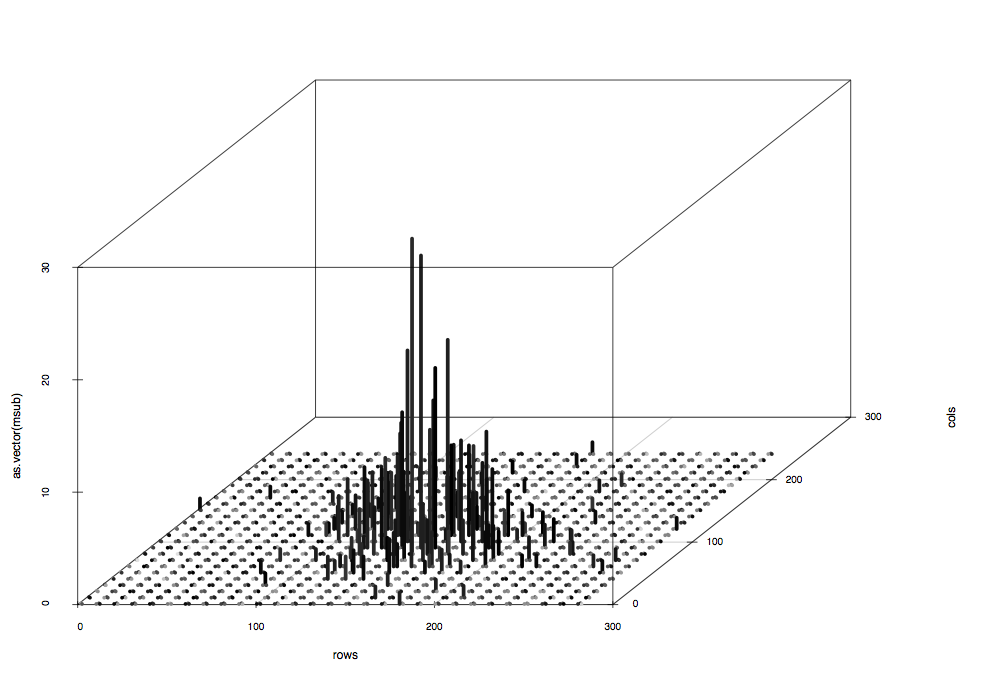


Figure 2. Histogram of simulated paternal contribution to a single ear. An enlarged area from a field of 60,000 plants is shown. Height of the bars indicates the number of times the same parent was represented in the seed. The blue arrow shows the location from which the ear was sampled.


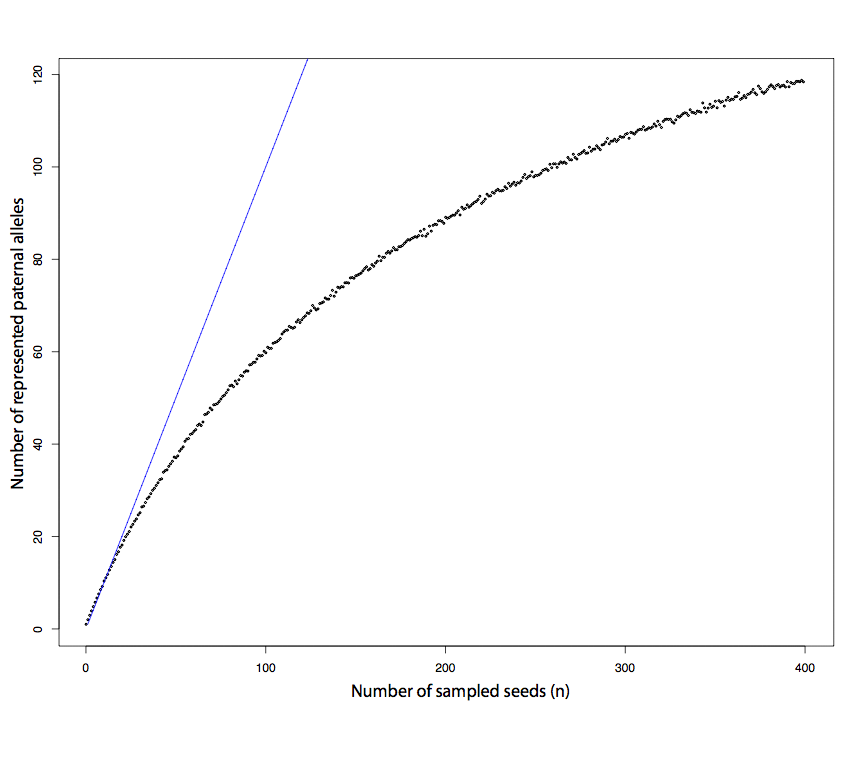


Figure 3. Estimated number of represented paternal alleles as a function of the number of sampled seeds. Blue line represents the expected number of alleles under unrestricted pollination.

**Expected detection probabilities for non-uniform frequency distributions.** When transgene frequencies differ between *m* different sampled fields, detection probability is calculated as:

(Lockwood et al. 2007)

For a sample of size$S$ taken from a single field selected at random and having an unknown transgene frequency $p_{i}$ we have:

Which for low values of $p_{i}$, may be written as:

or:

So that we have :

For *m* independently sampled fields we have:

(4)

When $Y=1$:

**
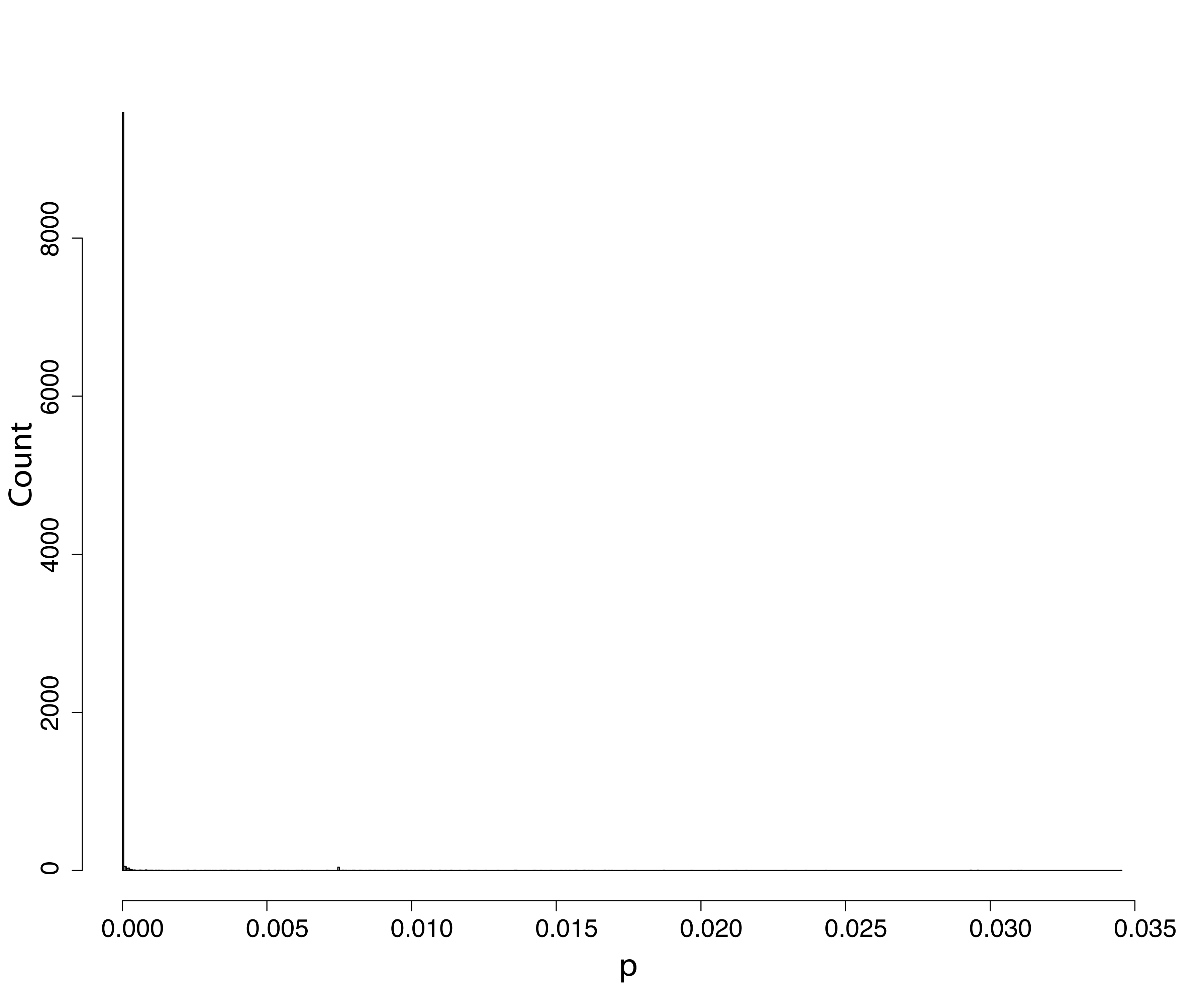
**

Figure 4. Histogram of allele frequencies observed in 10,000 random samples from 1000 simulated villages. It shows that transgene frequencies are expected to be highly skewed (compare Figure 2. in the main text).

**References:**

Cleveland, D. A., D. Soleri, F. Aragon Cuevas, J. Crossa and P. Gepts (2005). "Detecting (trans)gene flow to landraces in centers of crop origin: lessons from the case of maize in Mexico." Environmental Biosafety Research **4**(4): 197-208.

Crossa, J. (1989). "Methodologies for estimating the sample size required for genetic conservation of outbreeding crops." Theoretical and Applied Genetics **77**(2): 153-161.

Lockwood, D. R., C. M. Richards and G. M. Volk (2007). "Probabilistic models for collecting genetic diversity: comparisons, caveats, and limitations." Crop Science **47**(2): 861-868.

Ma, B. L., K. D. Subedi and L. M. Reid (2004). "Extent of cross-fertilization in maize by pollen from neighboring transgenic hybrids." Crop Science **44**(4): 1273-1282.

Messeguer, J., G. Penas, J. Ballester, M. Bas, J. Serra, J. Salvia, M. Palaudelmas and E. Mele (2006). "Pollen-mediated gene flow in maize in real situations of coexistence." Plant Biotechnology Journal **4**(6): 633-645.

Uribelarrea, M., J. Carcova, M. E. Otegui and M. E. Westgate (2002). "Pollen production, pollination dynamics, and kernel set in maize." Crop Science. Nov Dec **42**(6): 1910-1918.

Vencovsky, R. and J. Crossa (1999). "Variance effective population size under mixed self and random mating with applications to genetic conservation of species." Crop Science **39**(5): 1282-1294.
