# Supplementary figures and images for: Transgenes in Mexican maize: molecular evidence and methodological considerations for GMO detection in landrace populations
Source: Mol Ecol. 2009 Feb;18(4):750–61. doi: 10.1111/j.1365-294X.2008.03993.x (PMC3001031; doi:10.1111/j.1365-294X.2008.03993.x)

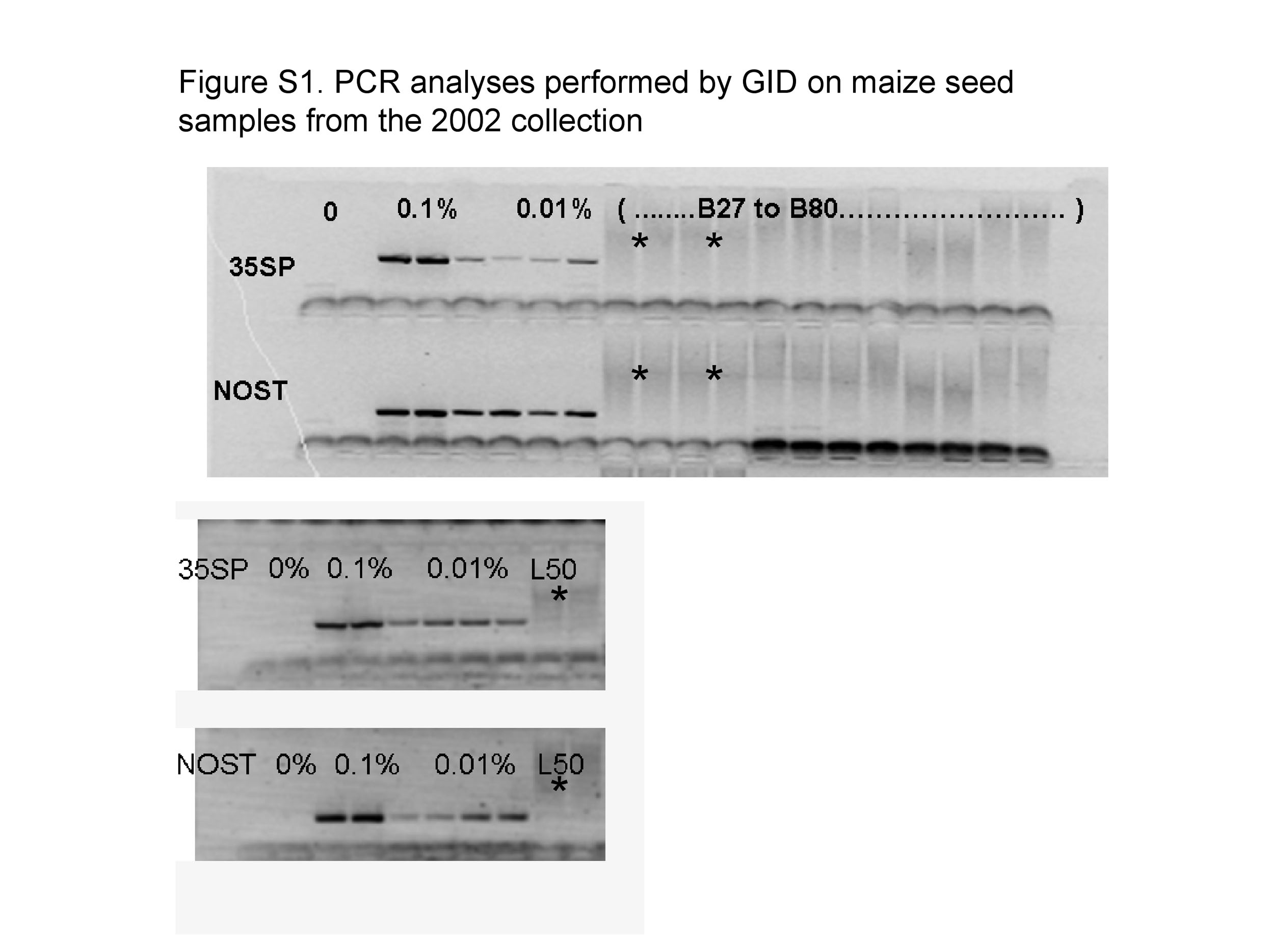

Supplement: Supplementary file 2 [file mec0018-0750-SD2.jpg]
