# Supplementary material for: Transgenes in Mexican maize: molecular evidence and methodological considerations for GMO detection in landrace populations
Source: Mol Ecol. 2009 Feb;18(4):750–61. doi: 10.1111/j.1365-294X.2008.03993.x (PMC3001031; doi:10.1111/j.1365-294X.2008.03993.x)
Supplement: Supplementary file 3 [file mec0018-0750-SD3.doc]

| **Table S1. Comparison of experimental conditions and results of the various experiments discussed or reported in this study** | | | | | | | | | | | | | |  |
| --- | --- | --- | --- | --- | --- | --- | --- | --- | --- | --- | --- | --- | --- | --- |
| ***Source*** | ***Sampling*** | | |  | ***Controls*** | |  | | ***Methods*** | |  | |  |  |
|  | **Years** | **Locations** | **Material** |  | **Negative** | **Positive** |  | **Analytical** | | **Corroboration** |  | **Results** | | |
| Quist & Chapela 2001, 002 | October-November 2000 | S.J.O: 4 standing fields + 1 store | 6 Whole ears |  | Peruvian maize  1971 collection from S.J.O. | Yieldgard (Bt) and RR-maize  *α-zein* |  | Ql PCR 35S/NOSt on DNA from bulked kernels | | DNA-DNA dot blot hybridization |  | 35S: in 5/7 samples; required nested PCR  NOSt: in 3/7 samples | | |
| Ortiz-García et al. 2005 | Nov.-Dec. 2003-2004 | S.J.O:  2003: 1-5 fields in 16 locations  2004: 16 locations (14 same, 2 new) | Whole ears or seeds  2003: 1 ear from 4-5 plants/field  2004: 10 plants-field: 5 normal + 5 stressed |  | PCR Buffer | Known amounts of 35S, NOSt; maize *Adh1* |  | Ql and Qn PCR: 35S/NOSt  2003: 50,126 seeds from 164 plants  2004: 103,020 seeds from 706 plants | | 2003: GID  2004: 1/3 GID; 1/3 GeneScan; 1/3 archived. |  | 35S/NOSt: none detected in both years  See text for frequency estimation details. | | |
| ***This study*** | |  |  |  |  |  |  |  | |  |  |  | | |
| (INE-CONABIO) | Fall 2001 | S.J.O: 21; Puebla state: 2 | Whole ears or seeds. 2 to 5 ears per farmer, one farmer per locality= 68 ears plus seed in 2 localities; Further PCR/SB analyses on selected samples |  | Peruvian maize; improved CIMMYT variety (BA),  Negatives from this collection,  PCR Buffer | DNA from NK603, MON810 and plasmid pMON18770  Maize HSP101 |  | IE-UNAM and CINVESTAV-I: Ql PCR for 35S-NOSt; sequencing of 35S | | GID: Ql and Qn  PCR 35S/NOSt  CINVESTAV-I, IE-UNAM: Ql PCR 35S/NOSt, 35S sequencing and 35S SB.(only UNAM) |  | UNAM/CINVESTAV: 35S in 3 Localities; (7, 11 and 23) ; 10 samples positive for 35S by PCR, 8 positive by SB.  35S sequences for selected samples  GID: all samples persent PCR amplification 35S/NOSt, 1 assigned as positive for the 35S (See fig 1 and Table 1) | | |
| Perales & Gepts | 2002 | S.J.O: 117-household survey over 5 locations | Whole ears: 9 ears/field or 6 stored ears/type. Blind-coded. Split samples for UCD and GID |  | DeKalb DKC 6215;  Historic sample: CIMMYT; no primers | Syngenta GSS 0966. FLUKA standards (based on MON810).  Maize invertase |  | Ql PCR on ground seed bulks | | Restriction digestion and sequencing of PCR products |  | 35S/NOSt: none detected in 120 samples | | |
|  |  |  |  |  | Peruvian maize improved variety from CIMMYT,  Buffer | Known amounts of 35S, NOSt target sequences; maize *Adh1* |  | Ql PCR on ground seed bulks | | IE UNAM: Ql PCR for the 35S on random leaf tissue bulks from this collection  Maize HSP101 |  | 35S/NOSt: none detected by GID on seed samples  35S: none detected by IE UNAM | | |
| Van Heerwaarden, Piñeyro, Perales &  Álvarez-Buylla | 2004 | S.J.O: :  2 localities (7 and 11 from 2001 collection) household survey | Individual leaf from standing maize plants; 300 leaves/field, 30 fields/locality; Analyses at IE- UNAM |  |  | DNA from NK603, MON810 and plasmid pMON18770;  Maize HSP101 |  | Ql PCR 35S/NOSt on: 50 leaf bulks  Maize HSP101 | |  |  | 35S: 3 fields in locality 11; 8 fields in locality 7.). | | |

Table S1. Comparison among different peer-reviewed studies conducted addressing the presence of transgenic sequences (35S and NOSt) in maize landrace samples from the Sierra Juárez, Oaxaca. Ql; qualitative PCR; Qn; quantitative PCR; SB: Southern Blot hybrization vs 35S promoter HSP101: maize Heat Shock Protein 101; 35S: 35S CaMV promoter; NOSt: Nopaline.synthase terminator Additional abbreviations as in Table 1. S.J.O: Sierra Juárez Oaxaca; GID: Genetic ID NA; UCD: University of California in Davis; CINVESTAV-I : Centro de Investigaciones Avanzadas unidad Irapuato; CIMMYT: Centro Internacional de Mejoramiento de Maíz y Trigo, México; IE-UNAM: Instituto de Ecología-Universidad Nacional Autónoma de México ; Additional information on other maize collections undertaken by Mexican governmental agencies and NGOs can be found in K. L. Mercer and J. D. Wainwright (2008)
