# Supplementary material for: Transgenes in Mexican maize: molecular evidence and methodological considerations for GMO detection in landrace populations
Source: Mol Ecol. 2009 Feb;18(4):750–61. doi: 10.1111/j.1365-294X.2008.03993.x (PMC3001031; doi:10.1111/j.1365-294X.2008.03993.x)
Supplement: Supplementary file 4 [file mec0018-0750-SD4.doc]

| **Table S2. Localities sampled in the 2001 Collection (INE-CONABIO).** | | | | | | | | |
| --- | --- | --- | --- | --- | --- | --- | --- | --- |
| Locality number and name | | State | Municipality | Altitude | Latitude | Longitude | Number of farmers | Number of ears |
| 1 | Carr. Palmarito-Tehuacán | Puebla | Palmar de Bravo | 1419 | 18°52’ | 97°38’ | 1 | 2 |
| 2 | Jesús Nazareno | Puebla | Palmar de Bravo | 2183 | 18°52’ | 97°37’ | 1 | 3 |
| 3 | Santa Maria Yahuiche | Oaxaca | Ixtlán de Juárez | 1806 | 17°17’ | 96°28’ | 1 | 3 |
| 4 | Santiago Comaltepec | Oaxaca | Santiago Comaltepec | 2028 | 17°33’ | 96°32’ | 1 | 3 |
| 5 | San Pablo Macuiltianguis | Oaxaca | San Pablo Macuiltianguis | 2153 | 17°32’ | 96°33’ | 1 | 3 |
| 6 | San Juan Analco | Oaxaca | San Juan Analco | 2138 | 17°24’ | 96°32’ | 1 | 5 |
| 7* | Santa Maria Jaltianguis | Oaxaca | Santa Maria Jaltianguis | 2074 | 17°21’ | 96°31’ | 1 | 6 |
| 8 | Rancho Tejas | Oaxaca | Ixtlán de Juárez | 2075 | 17°19’ | 96°28’ | 1 | 3 |
| 9 | Ixtlán de Juárez | Oaxaca | Ixtlán de Juárez | 2076 | 17°19’ | 96°29’ | 1 | 5 |
| 10 | Calpulalpan | Oaxaca | Calpulalpan | 2242 | 17°18’ | 96°26’ | 1 | 5 |
| 11* | Santiago Xiacui | Oaxaca | Santiago Xiacui | 2041 | 17°17’ | 96°26’ | 1 | 3 |
| 12 | Santiago Xiacui | Oaxaca | Santiago Xiacui | 2041 | 17°17’ | 96°26’ | 1 | 3 |
| 13 | La Trinidad | Oaxaca | Santiago Xiacui | 2035 | 17°15’ | 96°25’ | 1 | 3 |
| 14 | San Andrés Yatuni | Oaxaca | Santiago Xiacui | 2285 | 17°15’ | 96°24’ | 1 | 2 |
| 15 | Ixtlán de Juárez | Oaxaca | Ixtlán de Juárez | Almacén DICONSA | | | - | - |
| 16 | Ixtlán de Juárez | Oaxaca | Ixtlán de Juárez | Mercado local | | | - | - |
| 17 | San Juan Chicomezuchit | Oaxaca | Ixtlán de Juárez | 1806 | 17°17’ | 96°29’ | 1 | 2 |
| 18 | San Miguel Amatlán | Oaxaca | Ixtlán de Juárez | 2028 | 17°16’ | 96°28’ | 1 | 2 |
| 19 | Lachatao | Oaxaca | Pueblos Mancomunados | 2113 | 17°16’ | 96°28’ | 1 | 4 |
| 20 | El Punto | Oaxaca | Ixtepeji | 2422 | 17°13’ | 96°35’ | 1 | 2 |
| 21 | Las Presas | Oaxaca | Tlalistac | 1653 | 17°05’ | 96°39’ | 1 | 3 |
| 22 | Nochixtlán | Oaxaca | Nochixtlán | 1660 | 17°27’ | 97°13’ | 1 | 1 |
| 23* | Santo Tomás Teipan | Oaxaca  (Mixteca) | Santa María Ecatepec | 2380 | 16°15’ | 95°59’ | 1 | 5 |

Table S2. Localities sampled in the 2001 collection. Localities 1 and 2 are from the state of Puebla; localities 3 to 23 are from the state of Oaxaca; all localities sampled in the state of Oaxaca correspond to the Sierra Juárez region, except locality 23, which is in the Mixtec region, in the Southeastern part of the State. While we present here the original labeling provided by INE-CONABIO, it is important to note that localities 15 and 16 correspond to seed samples attained in stores in a single community, while localities 11 and 12 comprise the same community (Santiago Xiacui) and thus, can be taken as a single locality. In this case, there would be 20 localities with one farmer per locality (except in the case of Santiago Xiacui; labeled as localities 11 and 12) plus two additional seed sources (a DICONSA store (15) and an open market (16)). Number of participating farmers : 21, maize ears: 68. *: Localities positive for the 35S CaMV promoter (7, 11 and 23).
