# Supplementary material for: Transgenes in Mexican maize: molecular evidence and methodological considerations for GMO detection in landrace populations
Source: Mol Ecol. 2009 Feb;18(4):750–61. doi: 10.1111/j.1365-294X.2008.03993.x (PMC3001031; doi:10.1111/j.1365-294X.2008.03993.x)
Supplement: Supplementary file 5 [file mec0018-0750-SD5.doc]

| **Table S3. 2002 collection results. Localities and seeds samples analysed at GID and UCD are listed.** | | | | | | |
| --- | --- | --- | --- | --- | --- | --- |
| Locality | Community | Municipality | Seeds composing bulk sub-sample^a^ in GID | frequency 35S/NOSt | Seeds bulks  Analyzed at UCD^b^ | frequency 35S/NOSt |
| 1 | Chicomezúchitl parajes Vella/Tete/Xia | Ixtlán de Juárez | 2931 | 0 | 300 | 0 |
| 2 | Chicomezúchitl | Ixtlán de Juárez | 2098 | 0 | 350 | 0 |
| 3 | Tlalixtac de Cabrera | Tlalixtac de Cabrera | 2740 | 0 | 325 | 0 |
| 4 | Tlalixtac de Cabrera | Tlalixtac de Cabrera | 1183 | 0 | 250 | 0 |
| 5 | El Punto Ixtepeji | Santa Catarina Ixtepeji | 1233 | 0 | 175 | 0 |
| 6 | El Punto Ixtepeji | Santa Catarina Ixtepeji | 1507 | 0 | 350 | 0 |
| 7 | Santa María Yahuiche | Ixtlán de Juárez | 2165 | 0 | 225 | 0 |
| 8 | Calpulalpam | Calpulalpam | 332 | 0 | 100 | 0 |
| 9 | CONASUPO store | Tlalixtac de Cabrera | 154 | 0 | 50 | 0 |
|  |  | TOTAL number seeds | 14343 | 0 | 2975 | 0 |
|  |  | Average seeds/bulk^b^ | 5056.63 |  | 25 |  |

Figure S3. Localities sampled in the 2002 collection. While 9 localities are listed, localities 1 and 2, 3 and 4, as well as 5 and 6, are from the same community, and locality 9 corresponds to a DICONSA store. Thus, we sampled 5 communities (with two localities from three of them and one from the other two) and a DICONSA store. Sub-samples sent to GID and listed here represent seed lots from a particular locality. ^a^: These sub-samples were further bulked together at GID in order to comprise a sample. Samples analysed at UCD represented individual seed lots from a particular locality. For details regarding bulking schemes please refer to the Materials and Methods section at the main text. Note: Locality numbers used here do not correspond to the same codes used for localities/households collected in 2001 reported in Table S2. 35S; CaMV 35s promoter, NOSt; NOS terminator; Ql; qualitative PCR. ^a^:
